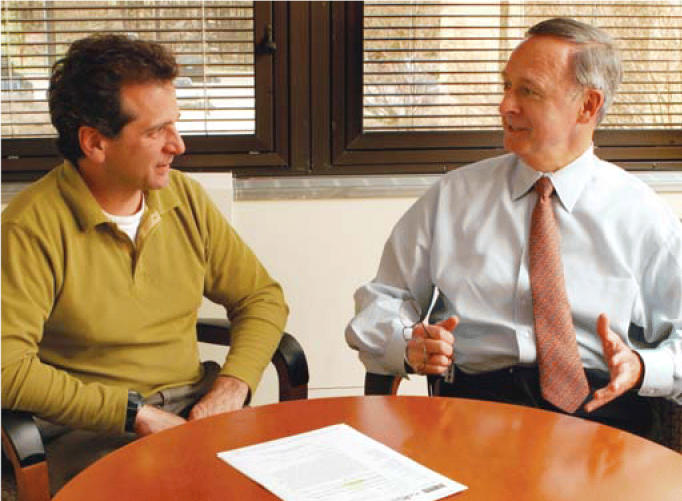# Translating Translational Biomedicine for Environmental Health

**DOI:** 10.1289/ehp.114-1440795

**Published:** 2006-04

**Authors:** David A. Schwartz, William J. Martin

**Affiliations:** Director, NIEHS and NTP, E-mail: david.schwartz@niehs.nih.gov; Director, Office of Translational Biomedicine, E-mail: wjmartin@niehs.nih.gov

“What’s in a name?” Shakespeare famously wrote. During our recent experience in conceptualizing and creating a new Office of Translational Biomedicine at the NIEHS, we have learned that the answer, often, is “quite a lot,” and perhaps necessarily so. The problem in this instance arises from the use of the term “translational.” This term has come to represent an area of biomedical research that, although full of promise, defies easy definition. Although variations of “translational medicine” and “translational research” are widely used to identify scientific programs in both public and private organizations, when asked what the term means, even many would-be practitioners might respond with some version of the “I know it when I see it” explanation. But it may be broadly described as using knowledge gained at the bench of basic research—a mechanistic understanding of disease—to improve clinical applications at the “bedside” or enhance disease prevention in the community. Our initial view was that the use of the term “translational” to describe this area of biomedical research is misleading, as the word carries many connotations, and is essentially inaccurate. However, this view is debatable.

The *Encarta World English Dictionary* defines translation as “the rendering of something written or spoken in one language in words of a different language.” The part of this definition that applies to the concept of translational biomedicine is the idea of different languages. Most basic researchers, clinical investigators, and public health scientists would agree that they speak essentially different “languages” when it comes to understanding and communicating the science of their respective fields. According to the NIH Roadmap for Medical Research, this communication gap is “limiting professional interest in the field and hampering the clinical research enterprise at a time when it should be expanding.” Perhaps nowhere is this more evident than in the disjunction between basic environmental health science research and the application of this knowledge to disease prevention, pathogenesis, and prognosis. While the reasons behind this are myriad, one of the current challenges for the NIEHS is to find a way to bridge this gap. This represents the fundamental challenge and opportunity of our new Office of Translational Biomedicine (whose name may yet change), which is now headed by William J. Martin II, a physician-scientist and former dean of the College of Medicine at the University of Cincinnati.

NIEHS scientists have contributed much toward understanding basic biology, genotoxicity, endocrine disruption, cell signaling, and oxidative stress, as well as to public health through understanding of morbidity and mortality of air pollution. Notable examples include elucidation of the effects of lead on IQ and the links between arsenic and cancer. While we will continue to strategically support these areas of investigation, we believe that we need to aggressively pursue new opportunities in translational biomedical research that simply were not available even a decade ago.

With the sequencing of the human genome, as well as the genomes of many model organisms, and the technological tools that are now becoming available, investigators can integrate knowledge gained through these advances into epidemiologic studies and bring fundamental biological approaches to the bedside. An example of this is the breakthrough in lymphoma research that now allows us to classify these diseases into distinct biological categories that have vastly different prognoses and responses to treatment. Similarly, investigators used this approach to understand the role of aflatoxin in the biological changes that lead to the development of liver cancer. Although this research took more than 30 years to complete, we can now more rapidly apply similar approaches to understanding how environmental exposures influence the basic biology leading to a number of diseases like asthma, reproductive disorders, atherosclerosis, diabetes, various types of cancer, autoimmune diseases, and neurodegenerative conditions. This requires basic scientists, clinical investigators, and public health scientists to find a new common language for describing disease mechanisms in order to improve human health. This is our opportunity and our responsibility.

[Translational biomedicine] requires basic scientists, clinical investigators, and public health scientists to find a new common language.

These efforts will be facilitated through the development of research initiatives that utilize and emphasize integration of basic, clinical, and public health sciences, development of interdisciplinary training programs, incorporation of a human disease–first approach to traditional environmental health sciences, and fostering of relationships among scientists of different disciplines to develop the research teams of tomorrow that will clearly delineate the central role of exposures in the pathogenesis of complex human diseases. If we are successful in these efforts, and we believe we will be, then the ultimate improvements in human health, by *whatever* name, will be a significant achievement.

## Figures and Tables

**Figure:**